# Help Is in Your Blood—Incentive to “Double Altruism” Resolves the Plasma Donation Paradox

**DOI:** 10.3389/fpsyg.2021.653848

**Published:** 2021-09-09

**Authors:** Petra Gyuris, Baksa Gergely Gáspár, Béla Birkás, Krisztina Csókási, Ferenc Kocsor

**Affiliations:** ^1^Institute of Psychology, Faculty of Humanities and Social Sciences, University of Pécs, Pécs, Hungary; ^2^Department of Behavioural Sciences, Medical School, University of Pécs, Pécs, Hungary

**Keywords:** medical fear, monetary reward, donor recruitment, altruism, plasma donation, COVID-19

## Abstract

Blood donation is considered as one of the purest forms of altruism. Plasma donation, in contrast, despite being a similar process, is mostly a paid activity in which donors are compensated for their contribution to the production of therapeutic preparations. This creates a so-called “plasma paradox:” If remuneration is promised for a socially useful effort, volunteers with altruistic motives might be deterred. At the same time, regular plasma donors who pursue the monetary benefits of donation might drop out if remuneration stops. The same controversy can be caught in the messages of most plasma donation companies as well: They promise a monetary reward (MR), and at the same time, highlight the altruistic component of donation. In this study, we tested the assumption that emphasizing the social significance enhances the willingness to donate blood plasma more effectively than either MR or the combination of these two incentives. This had to be rejected since there was no significant difference between the three scenarios. Furthermore, we also hypothesized that individuals might be more motivated to donate plasma if there is a possibility of offering an MR toward other socially beneficial aims. We found an increased willingness to donate in scenarios enabling “double altruism”, that is, when donating plasma for therapeutic use and transferring their remuneration to nongovernmental organizations, is an option. We propose relying on double altruism to resolve the plasma paradox, and suggest that it could serve as a starting point for the development of more optimized means for donor recruitment.

## Introduction

Medical therapeutic blood products, for instance, drugs made from blood plasma, play a crucial role in the health service enabling therapeutic interventions for both inheritable (e.g., anemia) and acquired (e.g., cancer) conditions (World Health Organization, 2020a). Therefore, there is an increasing demand for blood products (Lamb, 2009; Robert, 2009). From the perspective of supplying blood products, plasma donation, during which blood cells are returned into the body of the donor and only plasma is collected, has more advantages than blood donation; for instance, more products can be produced from a single donation, and it has fewer negative consequences for the donor (Ciavarella, 1992; Farrugia et al., 2015). Hence, it is of primary importance that governmental institutions and companies dealing with plasma donation should use effective methods to recruit donors. Sufficient amounts of blood and plasma are indispensable for the smooth running of health services; therefore, it is crucial to have a detailed picture of the nature of the donation activity.

### Blood Donation as a Form of Altruism

In popular thinking, blood donation is considered one of the purest forms of altruism. This is also reflected in the figures of global blood donations: The larger part of global supply, and in 62 countries, 100% of the collected blood arrives from nonremunerated donors (World Health Organization, 2020b).

Reference to moral norms is a common factor in the background of blood donation. In a previous study, 82% of blood donors and 85% of plasma donors reported that they donated to “do the right thing” (Glynn et al., 2001). Similarly, Godin et al. (2007) found that the internalization of personal values is decisive in the behavior of those who donate regularly. It is important to highlight that moral norms played a less important role in those who did not donate blood previously (Godin et al., 2005). These findings suggest that the development of an altruistic identity is essential for practicing regular blood donation (Piliavin and Callero, 1991).

Several researchers proposed that although remuneration, as an incentive, might be effective in the recruitment of young and first-time donors (Chell et al., 2018), it could also influence the motivation of regular donors in many unfavorable ways (Ariely et al., 2009). Money undermines the intrinsic motivation of those who donate regularly without being compensated and makes their role as altruists questionable, which they may experience as an attack against their personal identity (Masser et al., 2008). Accordingly, Costa-Font et al. (2013) proposed that incentives to blast donations should be used only to the extent that it does not counteract the altruistic identity of the donors.

It is also important to note that prosocial behavior is often driven by striving for a good reputation (Bereczkei et al., 2010). The fact that the frequency of donations can be increased with publicly announced (symbolic) prizes suggests that image concern also plays a significant role in blood donation (Lacetera and Macis, 2010). As monetary reward (MR) reduces the reputational value of a good deed achieved by blood donation, prosocial behavior may be partially, or even entirely, crowded out (Bénabou and Tirole, 2006), especially in public situations (Ariely et al., 2009). In relation to the crowding-out effect (Mellström and Johannesson, 2008), important sex differences were reported. While the willingness of women to donate whole blood decreased almost by half when an MR was introduced, it did not change for men. However, this crowding-out effect disappears, if the prospective blood donors have the opportunity to donate the money to a charity.

Although altruism plays an important role in blood and plasma donation, there are complex psychological processes in the background of donating behavior, of which the importance of good deeds represents only one factor. In previous studies, for example, self-efficacy (the belief that one can successfully perform a behavior) appeared to be the strongest predictor (Veldhuizen and Van Dongen, 2013), and convenience also seems to have a significant effect on donation. In a meta-analytic review, Bednall and Bove (2011) found that the most frequently cited motivator of both first-time (79.9%) and regular (80.1%) donors was the easy access to the blood collection site. In another review, the time required for the donation process, the inconvenient opening hours, and the location of the blood collection centers were identified as major determinants of decision-making (Piliavin, 1990) of the donors. Additionally, donation rates are influenced by economic forces, such as supply and demand (Slonim et al., 2014), but not associated with the volunteering rates, and show a wide cross-national variation that depends on the type of collection system in a given country (Healy, 2000). All of these results would be implausible if altruism was the sole motivation for donation behavior.

### Plasma Donation Paradox in Today's Health Service

Most plasma collection centers in the country in which the current study was performed seek to emphasize the MR and social benefits of donation behavior in their messages. For instance: “Money also comes for saving lives!”, “Save lives! Occasionally 8,000 HUF's, monthly up to 72,800 HUFs extra!” To obtain a more objective picture of this issue, we have taken 42 messages from the commercials, websites, and social media pages of plasma centers. The analysis confirmed our initial impression: Out of the eight plasma companies, three built their recruitment campaign solely on the money they could offer, and five on both money and the socially beneficial nature of the donation. Although altruism as a motivator appeared in single messages (websites, social media advertisements, etc.) as well, none of the companies use this incentive as the only means of donor recruitment (Table 1).

**Table 1:** The number of messages appeared in advertisements of plasma donation companies in the country being studied.

**Message focus**				
**Material goods offered**		**Social significance**	**Both**	**Total**
Money	20			
Prize	11			
Gift	2			
	33	7	1	42

Bove et al. (2011) found that most of the regular donors heard about plasma donations when they were unable to donate whole blood because of health reasons. Plasma donation was offered as a substitute for blood donation, and their first donation was made at the personal request of the staff. When nondonors were asked why they did not donate, the most common response was: “No one asked me personally” (Piliavin, 1990). Taking these findings into consideration, one effective way to recruit plasma donors is simply to contact the nondonors personally on behalf of the collection sites. If this is not possible, the combined use of phone calls and email reminders could also be effective (Germain and Godin, 2016).

However, little is known about the functioning of strategies to encourage plasma donation since the majority of the available research has examined the effectiveness of incentives in a nonremunerated blood donation context. Although there is an obvious need for continuous recruitment of plasma donors, very few studies have been devoted to the psychology of plasma donation (Charbonneau et al., 2015). Moreover, the motivations of blood and plasma donors differ even before the first donation (Veldhuizen and Van Dongen, 2013), so it is unclear whether the results of research on voluntary blood donors can be translated into country-specific remunerated plasma donation practices. In addition, although there are precedents for applying the results of a study directly by a blood collection center (Leipnitz et al., 2018), most researchers give advice to blood and plasma collection centers without testing the effectiveness of the proposed messages (Godin et al., 2005, 2007; Zhou et al., 2012). Taking into account that theoretical conclusions might not necessarily be utilized in real-life contexts, it would be particularly important in the future to test the effectiveness of advertising messages by allowing the collaboration of collection centers and researchers.

### Plasma Donation—Business or Altruism?

A large proportion of the global plasma supply is provided by paid U.S. donors (Lamb, 2009), raising the question of the importance of money in plasma collection. Despite extensive research into the relationship between MR and willingness to donate blood (Masser et al., 2008; Ariely et al., 2009; Costa-Font et al., 2013; Chell et al., 2018), much less is known about the interaction between plasma donation and financial incentives. Bove et al. (2011) found that plasma donors not only devaluate MRs for donations but also express concerns relating to nonmonetary incentives and suggest utilizing these resources to make the donation process more effective. It is important to note, however, that the aforementioned study examined the attitudes of plasma donors in a voluntary nonremunerated (VNR) environment. Indeed, Chell et al. (2018) pointed out that attitudes about different incentives also depend largely on the existing reward system. For example, Kretschmer et al. (2004) found that 86.1% of paid donors disagreed with the termination of the MR received for donation, and only 23% of the donors indicated that they would be willing to donate in the absence of financial benefits. Similarly, another study reported that 56.2% of plasma donors surveyed would be unwilling to continue donating without payment (Trimmel et al., 2005).

Similarly, in a sample of plasma-donating university students, Anderson et al. (1999) found that, regardless of their donation history, the primary motivation was money, which most of them (75%) spent on consumer goods (e.g., beer and cigarettes). The importance of money is also highlighted by the increase in Google searches for paid plasma donations during the global economic crisis in 2008 (Vasovic and DeSimone, 2019).

The following question arises: What happens if there is an attempt to convert paid donors into voluntary donors? To put it differently, is it possible to maintain the willingness of people to donate despite the fact that no MR will be given? In this area, there are only two old reports available. When payment for whole blood donation was phased out in New Mexico, USA, almost 100% of the paid donors stopped donating. This, nevertheless, had no effect on blood supplies, as blood collection center staff successfully recruited new voluntary donors (Surgenor and Cerveny, 1978). Although the conversion was unsuccessful, previous donors could be replaced with more altruistic ones who did not require compensation. In contrast, Grindon et al. (1976) reported a case when the conversion was successful. When the payments for blood had been eliminated in the Johns Hopkins Hospital, Baltimore, attempts were made to convert former professional donors to volunteers. Some of the previous donors (25%) had moved out of the area, and another 6% were ineligible for medical reasons. At the end of a 3-month campaign, 73% of the available donors, which is 49.5% of all previous donors, were successfully converted. However, this sample consisted of hospital workers. Hence, no clear conclusion can be drawn about the altruistic motives of whole blood donors. Concerning plasma donation, Bednall and Bove (2011) concluded in their meta-analysis that money is more important for plasma donors than for other donors.

Therefore, the question is: What factors explain the difference in motivation between blood and plasma donors? Given that 97% of the blood collected in the European region comes from unpaid donors (World Health Organization, 2020a), and in a paid context, MRs are more important for donors than for those who have never donated (Tscheulin and Lindenmeier, 2005). This suggests that people can also be motivated by nonfinancial incentives to donate. Considering that plasma centers place a significant emphasis on the MRs for donation (see Table 1), it is conceivable that this marketing strategy will omit a separate potential donor base that could be motivated to donate for altruistic reasons as well. It is known that MRs do not crowd out prosocial behavior if donors are given the opportunity to use the money received for charitable purposes (Mellström and Johannesson, 2008). Therefore, it is possible that using an incentive we call “double altruism”, many new donors could be recruited if plasma centers would provide the opportunity to transfer the MR they receive to charities, thus creating a new form of altruism that is also compatible with payments.

### Aims and Hypotheses

In recent decades, several studies have been conducted to investigate the psychology of plasma donation, identifying many motivators, such as the perceived need for donation, high self-efficacy, and prosocial behavior (Bednall and Bove, 2011), as well as deterrents such as vasovagal reactions, the time required for donation, and fear of contamination (Beurel et al., 2017). Although many determinants are identified in the scientific literature, no motivational strategy has been found so far toward which both donors and nondonors would have a positive attitude, and would not have a negative impact on blood safety. Since money, as a primary motivation, can attract more at-risk individuals, the rate of unusable donations can be high (e.g., due to the harmful substances or infectious agents in the collected blood) and could negatively impact the quality of the collected blood (Chell et al., 2018). The aim of this research is to examine whether the currently widespread strategy of dual messages provides an optimal motivational background for potential plasma donors. More specifically, we examined the separate and joint effects of MR and emphasizing the social significance (SS) of the altruistic act on donation willingness. Furthermore, we also tested whether converting MR into another act of donation [by offering participants the possibility to transfer the received compensation to a nongovernmental organization (NGO)] might increase donation willingness as well. Based on the above literature, we phrased the following hypotheses:

1 Participants who read about the social benefits of plasma donation will report a greater willingness to donate than those who read about receiving an MR.
2 If the SS is emphasized, mentioning an additional MR will reduce the willingness to donate.
3 Those who have the opportunity to transfer the MR to an NGO will report a higher willingness to donate than those who receive only the MR.
4 Those who have the opportunity to transfer the MR and also read about the social benefits of plasma donation will report a higher willingness to donate than those who only have the opportunity to transfer the MR.

## Materials and Methods

### Participants

We recruited participants through social media and the mailing list of the University of Pécs, Hungary. In total, 333 subjects (mean age = 33.3, SD = 14.5, range = 19–77 years; 82 women, 250 men, 1 androgynous individual) completed the online survey at least to the point where the question related to their willingness to donate blood plasma was presented. This number includes 15 participants who did not finish the final questions of the survey; however, we retained their responses in the dataset.

### Procedure

Participants completed the survey online, using Psytoolkit (Stoet, 2010, 2017). First, they were asked to report demographic data, followed by questions about knowledge of, experience with, and attitude to, blood and plasma donation (see Supplementary Material). After that, participants were assigned randomly to one of five groups. In each group, having read a short scenario, they were required to report the likelihood with which they would donate plasma, using a slider. Their answers were scored from 0 (very unlikely) to 100 (very likely). There was no feedback on the scores they gave; participants had to rely purely on the graphical interface. The scenarios emphasized different aspects of the donation, including the monetary benefits and the social usefulness of their act—namely, the therapeutic use of blood plasma—, and the combinations of these. The scenarios were the following:

1 MR
2 SS
3 MR and SS
4 MR that can be donated to an NGO
5 MR donated to an NGO and emphasizing SS.

Before answering the last two scenarios, participants who were assigned to these were asked: What is the profile of the NGO they would most likely support: animal welfare, support of children, or the support of people living in poverty or who are homeless? The text of the actual scenario was adapted to their responses. For instance, if someone was randomly assigned to the NGO and SS scenario and indicated their preference to support children, they were exposed to the following text: “Suppose that a new plasma center opens not far from your home. Life-saving medical products are made from the plasma collected in this center and the donors receive 8,000 HUFs for each plasma donation which they can offer to the SOS Children's Villages, Hungary, an NGO providing loving and safe homes to children who can no longer live with their families. How likely do you think you would donate plasma? Indicate your answer using the slider!” In other scenarios, some of the above information was omitted (e.g., reference to money, NGO, medical products, etc., respectively. Refer Supplementary Material for detailed descriptions of the scenarios).

### Control Variables

#### Health Status

Participants were also asked to answer some questions about their health. If they did not wish to answer, they were free to move to the next question. First, they had to report on a five-point Likert-scale (from *very bad* to *excellent*) their own subjective evaluation of their perceived health status in comparison with other people of their age. The following questions were related to their chronic and mental conditions, and, if any, the length of time that they had suffered from it. Lastly, participants were asked how many times they had visited any type of health care facility (general practitioner, specialist, screening, etc.).

#### Medical Fear Survey

The short version of the Medical Fear Survey (MFS-short; Olatunji et al., 2012) is a 25-item self-report measure to assess the fear of medical treatments and related factors through five dimensions including fears of injections and blood draws, sharp objects, blood, mutilation, medical examination, and physical symptoms. Participants rate their degree of fear on a four-point Likert-scale (0–3) referring to the intensity of their fear if they were exposed to medically related situations described by the items. Cronbach's alphas of the subscales were 0.89 (*injection and blood draws*), 0.82 (*sharp objects*), 0.81 (*examination and symptoms*), 0.9 (*blood*), and 0.84 (*mutilation*), whereas reliability values in our sample were 0.86, 0.82, 0.79, 0.87, and 0.83, respectively.

#### Knowledge of, Experience With, and Attitude Toward Plasma Donation

People who partook in the study were asked whether they had donated blood or plasma previously, how many times, and how many months ago the last donation was. About half of all participants have already donated blood, but only 14% had experience with plasma donation. About 80% of the latter were previously blood donors as well, whereas only 23% of blood donors had experience with plasma donation. Hence, plasma donors are underrepresented both in the whole sample and among the blood donors (Table 2).

**Table 2:** Contingency table of participants according to their previous experience with blood and plasma donation.

**Blood donation**		**Plasma donation**		
		**Yes**	**No**	**Total**
Yes	Observed	37	125	162
	% of total	11%	38%	49%
No	Observed	10	161	171
	% of total	3%	48%	51%
Total	Observed	47	286	333
	% of total	14%	86%	100%

To the best of our knowledge, there are no validated questionnaires that aim to assess the depth of information available to donors about plasma donation. Therefore, we used a modified version of a questionnaire that measures knowledge about blood donation (Renzaho and Polonsky, 2012). The modification consisted of simply changing the word “blood donation” to “blood plasma donation” and changing the scoring accordingly. The reliability of the original scale was 0.783 (Kuder-Richardson Reliability Coefficients—KR20), whereas that of the modified version was much lower, 0.592. This suggests that the knowledge of participants about the requirements, procedure, and benefits of plasma donation is less consistent than that regarding blood donation. However, since actual knowledge could potentially affect willingness to donate plasma, we kept the scores on the scale as one of the control variables.

We also asked participants to report what they think about plasma donation. They were required to give their ratings on a slider from −3 to +3 to six item pairs: Plasma donation is (1) unpleasant—pleasant, (2) ethically questionable—creditable, (3) selfish—altruistic, (4) pointless—helpful for a lot of people, (5) stressful—calming, and (6) useless—useful. Apart from the position of the slider, there was no feedback on the scores they gave.

#### Questions Related to Coronavirus

As the survey was run during the peak period of the first wave of the coronavirus disease (COVID)-19 crisis in Hungary, we included some control questions about the knowledge of participants about and attitude toward the coronavirus. Most importantly, an injection of blood plasma from healed patients appears to be an effective therapy in treating patients (Malani et al., 2020). As awareness of this could potentially influence attitude toward plasma donation, we asked participants to indicate whether they heard of this treatment method. Furthermore, based on the suggestions of the World Health Organization (2020c), four other questions were asked to which they had to answer by moving a slider. The left side indicated “not at all” (1 point), and the right “extremely” (10 points). The questions were related to worry about becoming infected, concern for family members, depression from news, and receiving support from family and friends.

#### Statistical Analyses

Descriptive analyses and the ANOVA were carried out in Jamovi 1.1.9.0 (The Jamovi Project, 2019), the Generalized Linear Mixed Model (GLMM) was made using SPSS 26.0, and the power analysis with G-power. Given that the distribution of the variable in focus, that is the score indicating the willingness to donate blood plasma, was not normal (Shapiro-Wilk test, W = 0.946, *p* < 0.001), we used robust methods that do not assume normality of the data, where possible. Levene's test of equality of variances was not significantly different between the five scenarios (F = 1.57; df = 4, 328; *p* = 0.181); therefore, in all of the analyses, we assumed that variances are equal.

## Results

### Differences in the Willingness for Plasma Donation Between the Scenarios

To test whether there was a difference between the answers given in the five scenarios, we used Fisher's one-way ANOVA. The results show that globally there is a significant difference between scenarios (F = 4.34; df = 4, 328; *p* = 0.002, f = 7.95, 1–β = 1.00. Refer also Supplementary Material for the details of the power analysis). Tukey's *post-hoc* analysis revealed that the difference in donation willingness was significantly higher in the NGO and the NGO and SS scenarios than in the MR scenario. The mean score of the NGO scenario was higher than that of the MR and SS scenario (refer Table 3 for descriptives and Table 4 for *post-hoc* test results).

**Table 3:** Group descriptives of the donation willingness scores in the five conditions used in the ANOVA.

**Condition**	***N***	**Mean**	**SD**	**SE**
MR	71	41.0	35.4	4.20
SS	60	50.5	37.0	4.77
MR and SS	74	44.8	36.0	4.19
NGO	64	62.0	33.7	4.21
NGO and SS	64	58.2	31.5	3.94

**Table 4:** Tukey's *post-hoc* test results of Fisher's one-way ANOVA made on the donation willingness scores.

		**MR**	**SS**	**MR and SS**	**NGO**	**NGO and SS**
MR	Mean difference	–	−9.54	−3.76	−20.99**	−17.19*
	*t*-value	–	−1.56	−0.65	−3.50	−2.87
	df	–	328.00	328.00	328.00	328.00
	*p*-value	–	0.522	0.967	0.005	0.036
SS	Mean difference		–	5.78	−11.45	−7.65
	*t*-value		–	0.96	−1.83	−1.22
	df		–	328.00	328.00	328.00
	*p*-value		–	0.874	0.357	0.737
MR and SS	Mean difference			–	−17.23*	−13.43
	*t*-value			–	−2.90	−2.26
	df			–	328.00	328.00
	*p*-value			–	0.032	0.160
NGO	Mean difference				–	3.80
	*t*-value				–	0.62
	df				–	328.00
	*p*-value				–	0.972
NGO and SS	Mean difference					–
	*t*-value					–
	df					–
	*p*-value					–

* *p <0.05*,

** *p <0.01*.

### Effects of Control Variables on Plasma Donation

To test whether any of the control variables contribute to the willingness to donate blood plasma, we built a GLMM (Laird and Ware, 1982). We used *donation willingness* as the target variable with normal probability distribution and identity as a link function. Categorical variables were compared using sequential Bonferroni correction. We set the following variables as predictors (variable names in italics):

*Sex* of participants (binary variable). Only men and women were retained in the sample.

*Age* of participants (continuous variable)

Previous experience with *blood donation* (categorical variable)

Previous experience with *plasma donation* (categorical variable)

*Excluded* from plasma donation because of a chronic condition or other reasons (categorical variable)

*Health status* (continuous variable)

Number of times the participant visited a *health care* institution in the last 12 months (continuous variable)

Scores on the scale assessing *knowledge* about plasma donation (continuous variable)

*Attitude* toward plasma donation (continuous variable)

*Condition* to which the participant was assigned (categorical variable)

Scores on the five *MFS subscales* (continuous variable)

The model was significant (F = 9.435, df1 = 19, df2 = 294, *p* < 0.001), and the value of the Akaike Information Criterion was 2,928.491, which increased when we tried to omit any nonsignificant fixed effects. This suggests that important information would be missing from the model if any of the variables (even one with a low significance value) was dropped. The significant predictors of donation willingness were *age, blood donation* and *plasma donation* experience, *attitude* toward plasma donation, test *condition*, and the *MFS injection* subscale. The fixed coefficients show that *attitude* was associated positively (coeff. = 2.142), whereas *age* (coeff. = −0.460) and fear of *injection* (coeff. = −2.402) negatively with donation willingness. Those who had previous experience with blood donation and plasma donation scored on an average 12.785 and 15.021 points higher on the donation willingness scale than those who did not, respectively. The fixed coefficient of the variable *condition* echoed the results of the ANOVA, with participants scoring significantly higher in conditions with the possibility of offering MR to NGOs than in the others (Refer Supplementary Material for detailed statistics).

### Control Questions Related to Coronavirus

Of the 320 participants who have completed the test until this point, 59 reported that they were not aware of the potential use of blood plasma as a treatment method for overcoming COVID-19. Therefore, we ran a Welch's independent samples *t*-test on the donation willingness scores, which indicated that there was no significant difference between the two groups in any of the five scenarios (Supplementary Table 1).

The descriptive statistics of the questions suggested by the World Health Organization (2020c) (for which responses between 1 and 10 could be given with a slider) showed that participants were moderately concerned about possible infection. The mean score, which shows how much they are worried for their relatives, is slightly above the theoretical midpoint, the depression from news scores, and the total of the previous three questions were close to the midpoint. They reported receiving substantial support from family and friends (Supplementary Table 2).

## Discussions

Currently, all of the available means and strategies that try to spur plasma donation have some disadvantages (Chell et al., 2018). The aim of the current research was to map the attitude of potential donors toward different marketing strategies, and their previous experiences with plasma donation, in association with their fear of medical interventions, age, health status, and knowledge of plasma donation. We investigated the donation willingness of participants using a between-subjects study design. In the first two hypotheses, we assumed that emphasizing SS enhances the willingness to donate blood plasma more effectively than either MR or the combination of these two incentives. This had to be rejected since there was no significant difference between the SS, MR, and SS and MR scenarios. This result contradicts previous studies showing that some donors might change their minds when monetary compensation is offered (Masser et al., 2008). Furthermore, the exact amount of the MR is crucial. We would like to highlight that we tested the separate and joint effects of social motivations and typical MRs only, rather than the optimal sum that would be the most effective in an economic sense to attract donors. Therefore, we used a sum that is offered by the majority of plasma donation companies in the home country of the participants. Though there might be no differences between the probability of donation willingness in the SS, MR, and SS and MR scenarios in the current study, the number of actual donors that are sensitive to one or the other message would have a huge impact on the effectiveness of the total amount of collected blood. However, our survey did not try to catch the different motivations of the participants that might influence their responses in the specific conditions. There might be subpopulations in the sample that differ in their altruistic motives. Since the identification of the target population is of crucial importance when designing an effective marketing strategy, these potential idiosyncratic differences should be addressed in future studies.

Nevertheless, in accord with the third and fourth hypotheses, we found that those potential donors who have the opportunity to immediately forward the received monetary compensation reported a higher willingness to donate plasma than those who simply received the money. This result echoes other recent publications. First, it is known that people can be motivated to donate without monetary incentives since there are countries where the necessary blood supply is ensured by nonremunerated donations (World Health Organization, 2020b). Second, although MR might repulse some individuals from donating (Masser et al., 2008), a study on whole blood donation showed that their willingness to donate would not change if they have the option of offering the money to NGOs (Mellström and Johannesson, 2008). However, it is uncertain whether this effect might be present in the case of plasma donation as well. Plasma apheresis, in contrast to whole blood donation, is a lengthy and physically more demanding procedure that also requires regular involvement of the participants. Though our study confirmed the hypothesis that the option to offer payment to NGOs increases the willingness to donate, we still are in need of a field study that repeats this effect on the behavioral level as well.

Contrary to the assumption of our fourth hypothesis, NGO and NGO and SS conditions did not differ significantly. One of the reasons for the lack of difference might be that because the pure NGO scenario could already stimulate the feeling of double altruism, donation willingness of participants could reach a ceiling effect that would not be exceeded by the additional information of social usefulness.

The results imply that donation willingness could be enhanced significantly in that case only when *double altruism* is an option for the potential donors. However, it is still possible that the first two hypotheses had to be rejected because of the institutional background of plasma donation in the country where the research was conducted. In Hungary, plasma donation happens exclusively as a paid contribution. It is possible, even in the scenarios when only social usefulness was emphasized, that mentioning plasma donation evoked the concept of a remunerated donation in the participants, offsetting the first three conditions. Further cross-cultural research is needed to shed light on this issue.

The GLMM, which we used to control for the factors that might influence donation willingness, showed that attitude toward and experience with blood plasma and whole blood donation are significantly and positively associated with donation willingness. Previous research has already shown that moral norms are important for blood donors (Godin et al., 2005), therefore, they might more easily be recruited in other prosocial acts. The results might indirectly reflect a similar attitude of plasma donors, however, as all of the conditions, except the SS scenario, included MR, it is impossible to disentangle whether the primary motive was to help or to earn money. Nevertheless, it is not yet clear what causes the higher donation willingness of previous blood and plasma donors: Is their higher level of altruism an inherent part of their personality, or is it a change in their personal norms caused by their previous experience? It has been shown that moral nudges can promote prosocial behavior (Capraro et al., 2019; Capraro and Perc, 2021). For instance, those who take part in an act of donation are exposed to people who follow a social norm that is altruistic, and this could shift their own personal moral preferences as well. An altruistic identity is essential for regular blood donors (Piliavin and Callero, 1991), and the same might be the case in situations when SS of plasma donation is emphasized.

The GLMM also showed that if age is higher, the willingness decreases, but there was no detectable connection between willingness and health status or prevalent chronic diseases. This might be counterintuitive in light of previous research that pointed out that low self-efficacy is a negative predictor of contributions (Beurel et al., 2017), and that health problems are likely to negatively influence the perceived ability to give blood or plasma. As a note of caution, when mentioning the thwarting effect of health problems, previous research focused mostly on vasovagal reactions (France et al., 2004; Newman et al., 2006; Amrein et al., 2012; Bagot et al., 2013), and this is also echoed by the MFS *injection* subscale's contribution to the model. In summary, the control variables did not change the overall picture, as the condition to which the participants were assigned remained a significant predictor of donation willingness.

The survey included a question about the awareness of participants about the fact that an injection of plasma from healed COVID-19 patients might be an effective therapy for the treatment of patients with severe symptoms (Malani et al., 2020). As the survey was started in the middle of the coronavirus pandemic, this information could influence altruistic behavioral motives of people toward plasma donation, and could therefore be detrimental for the generalizability of the results, if not controlled for. The result that scores of donation willingness were independent of knowledge of participants about the potential use of plasma in an ongoing epidemic suggests that the results are not artifacts generated by a special situation, but rather reflect a general pattern of reactions given to different messages of plasma donation agencies.

## Limitations

It is important to note that our research also has some limitations. The first and most important aspect to emphasize is that our study examined the attitudes, and not the actual behavior, of potential donors toward different marketing strategies, thus raising the question of how the results can be transferred into practice. That said, there is some evidence that whole blood donation behavior correlates positively with donation intentions, and to a lesser extent with attitudes as well (Bagot et al., 2013). Cognitive and affective attitudes toward donation were found to be positively associated with intentions to donate in both first-time whole blood and plasma donors. Having a high level of intention, in turn, increases the odds of becoming a plasma donor (Veldhuizen and Van Dongen, 2013). However, answering the question as to whether the attitude measured in the NGO scenarios of the current study predicts donation behavior indeed, goes beyond the scope of the current research. Future studies should address not only the attitudes but also follow-up on the donation behavior of subjects after being exposed to specific messages.

Another potential limitation comes from the demographic distribution of the sample. Fifty-five percent of the participants indicated that they are students. Although students of higher education institutions represent a crucial basis for plasma donation (Anderson et al., 1999), it is possible that different age groups can be reached, and motivated to donate, using different messages. Similarly, although the proportion of men is higher among blood donors (Healy, 2000), women are disproportionately underrepresented in our sample. To the best of our knowledge, no study with a representative sample has yet been published about the demographic distribution of plasma donors. The statistical analyses in the current study suggest that older people tend to have a lower willingness to donate blood plasma, and men and women do not differ in their donation willingness.

Besides, the study population consists of 14% previous plasma donors. Though this also means that our findings are not generalizable to the whole population, in fact, we intended to test whether there is a way to reach potential plasma donors who are not already recurring visitors at plasma centers. The obvious differences between attitudes of successfully recruited and potential donors do not mean that with messages phrased to fit the expectations of specific populations, donation willingness could not be triggered to a different extent. Finding appropriate messages would be decisive in transforming research into practice.

## Conclusions

This study aimed to map the attitude of people toward different messages that reach them from plasma donation agencies, in association with their fear of medical interventions, and previous experience with, and attitude toward, donation. As the demand for blood products is increasing year by year worldwide (Lamb, 2009; Robert, 2009), it is critical for plasma centers to be able to use effective strategies to recruit donors.

The main assumption was that the marketing strategy of plasma agencies that emphasize monetary compensation is at least partly responsible for the motivational difference between blood and plasma donors (Bednall and Bove, 2011). Therefore, potential plasma donors might be more motivated by messages that highlight and multiply the social usefulness by giving the possibility to immediately transfer the remuneration to charitable organizations. The results support this idea. We called this phenomenon double altruism, referring to the behavior when people act altruistically twice: First with their blood plasma, and second by giving away the compensation received. The results highlight the potential of developing and testing specific advertising messages that set aside the disadvantages of strategies that rely either on MR (Masser et al., 2008) or pure altruism (Surgenor and Cerveny, 1978; Kretschmer et al., 2004; Trimmel et al., 2005), hence resolving the plasma paradox and creating a more optimized means for donor recruitment.

## Data Availability Statement

The datasets presented in this study can be found in online repositories. The names of the repository/repositories and accession number(s) can be found below: https://bit.ly/double_altruism.

## Ethics Statement

The studies involving human participants were reviewed and approved by United Ethical Review Committee for Research in Psychology, Hungary. The patients/participants provided their written informed consent to participate in this study.

## Author Contributions

PG, BGG, BB, KC, and FK conceived the study design. BGG and FK wrote the draft of the manuscript. FK and BB discussed the statistical analyses, and FK carried out the analyses. BGG and FK contributed to data collection and data processing. PG, KC, and BB helped to prepare the final version of the manuscript. BGG finalized the layout and proofread the manuscript. All authors contributed to the article and approved the submitted version.

## Funding

This work is financially supported by the European Union, co-financed by the European Social Fund (EFOP-3.6.1.-16-2016-00004—Comprehensive Development for Implementing Smart Specialization Strategies at the University of Pécs). BGG was supported by the ÚNKP-2020 New National Excellence Program of the Ministry for Innovation and Technology from the source of the National Research, Development, and Innovation Fund, and by the Ildikó Kriszbacher Scholarship for Talents of the University of Pécs, Hungary.

## Conflict of Interest

The authors declare that the research was conducted in the absence of any commercial or financial relationships that could be construed as a potential conflict of interest.

## Publisher's Note

All claims expressed in this article are solely those of the authors and do not necessarily represent those of their affiliated organizations, or those of the publisher, the editors and the reviewers. Any product that may be evaluated in this article, or claim that may be made by its manufacturer, is not guaranteed or endorsed by the publisher.
