# Supplementary material for: Help Is in Your Blood—Incentive to “Double Altruism” Resolves the Plasma Donation Paradox
Source: Front Psychol. 2021 Sep 9;12:653848. doi: 10.3389/fpsyg.2021.653848 (PMC8458749; doi:10.3389/fpsyg.2021.653848)
Supplement: Supplementary file 1 [file Data_Sheet_1.PDF]

Supplementary Material

SM 1 Questions related to knowledge of plasma donation

Please indicate whether the statement is wright or wrong.

1. Plasma donors get a medical exam before every plasma donation.
2. Plasma donors have to weigh at least 50 kg.
3. Plasma donors are tested for blood-based infections, like HIV and Hepatitis.
4. Plasma donors' iron levels are checked before every donation.
5. People under the age of 16 are allowed to donate plasma.
6. Most patients needing plasma require donations from many people.
7. If there is a shortage in donor plasma in a country, this can be easily imported from another country.
8. Hungary exports a large amount of plasma to other countries.
9. Plasma is only given to people who can afford to buy it.
10. Plasma has to be used within 24 hours of donation.
11. It takes more than 90 minutes to donate plasma.
12. Plasma donors might get infections from the needles used to collect plasma.
13. One can donate plasma every 2-3 months.
14. People in Hungary receive payments for donating plasma.
15. If someone or their family members donate plasma, then they are entitled to get it for free.
16. Within 24-48 hours after donation plasma donors' blood volume is restored.

SM 2 Questions related to the experience with blood and plasma donation

1. Have you ever donated blood?
2. Approximately how many times have you donated blood?
3. How many months ago was your last blood donation?

4. Have you ever donated plasma?
5. Approximately how many times have you donated plasma?
6. How many months ago was your last plasma donation?

7. Do you plan to donate plasma within a year?
8. Would you advise your acquaintances to donate plasma?

9. *Please indicate your opinion of plasma donation using the slider.*

unpleasant – pleasant
morally questionable – morally virtuous
selfish – selfless
pointless – helps a lot of people
stressful – calming
useless – useful

SM 3 Scenarios which the participants were assigned to

1

Suppose that a new plasma center opens not far from your home, where the donors receive 8,000 HUFs for each plasma donation. How likely do you think you would donate plasma? Indicate your answer using the slider!

2

Suppose that a new plasma center opens not far from your home. Life-saving medical products are made from the plasma collected in this center. How likely do you think you would donate plasma? Indicate your answer using the slider!

3

Suppose that a new plasma center opens not far from your home. Life-saving medical products are made from the plasma collected in this center and the donors receive 8,000 HUFs for each plasma donation. How likely do you think you would donate plasma? Indicate your answer using the slider!

4

Suppose that a new plasma center opens not far from your home. The donors receive 8,000 HUFs for each plasma donation which they can offer to the Noah's Animal Home Foundation, which currently

serves as the largest animal shelter in Hungary for more than 1200 animals that were abandoned, tortured, or were victims of an accident. How likely do you think you would donate plasma? Indicate your answer using the slider!

5

Suppose that a new plasma center opens not far from your home. Life-saving medical products are made from the plasma collected in this center and the donors receive 8,000 HUFs for each plasma donation which they can offer to the SOS Children's Villages, Hungary, an NGO providing loving and safe homes to children who can no longer live with their families. How likely do you think you would donate plasma? Indicate your answer using the slider!

SM 4 Post-hoc power analysis output of G-power

F tests – ANOVA: Fixed effects, omnibus, one-way
Analysis: Post hoc: Compute achieved power

**Input:**	Effect size f	=	7.94665
	α err prob	=	0.05
	Total sample size	=	333
	Number of groups	=	5
**Output:**	Noncentrality parameter λ	=	21028.7
	Critical F	=	2.3991789
	Numerator df	=	4
	Denominator df	=	328
	Power (1-β err prob)	=	1.0000000

SM Table 1. Welch's independent samples t-test of subjects who were aware or not aware of the potential therapeutic use of plasma

Condition	Welch's t	df	p	Mean difference	SE difference	Cohen's d
Donation willingness (all conditions)	1.5824	84.55	0.117	8.197	5.18	0.2323
MR	1.4748	9.35	0.173	18.121	12.29	0.5162
SS	1.3364	11.34	0.208	17.632	13.19	0.4792
MR&SS	1.7888	19.91	0.089	19.153	10.71	0.5445
NGO	0.0530	11.96	0.959	0.665	12.55	0.0197

Condition	Welch's t	df	p	Mean difference	SE difference	Cohen's d
NGO&SS	-0.1753	33.53	0.862	-1.495	8.53	-0.0470

SM Table 2. Descriptives of scores on questions related to coronavirus

	Fear from infection	Concerned about family	Depression from news	Total anxiety	Received support
N	318	318	318	318	318
Missing	15	15	15	15	15
Mean	3.97	6.96	5.01	15.9	7.74
SD	2.60	2.55	2.50	6.24	2.46

The tables below refer to the GLMM that was built to analyse the effects of the control variables on the donation willingness scores.

SM Table 3. Fixed effects of the independent variables

**Fixed Effects**[a]	F	df1	df2	p
Corrected Model	9.435	19	294	.000
Sex	1.074	2	294	.343
Age	12.759	1	294	.000
Blood donation	12.284	1	294	.001
Plasma donation	8.515	1	294	.004
Excluded	1.250	1	294	.264
Health status	.026	1	294	.872
Healthcare	.120	1	294	.729
Knowledge	.385	1	294	.536
Attitude	56.344	1	294	.000
Condition	5.295	4	294	.000
MFS injection	9.270	1	294	.003
MFS sharp objects	3.074	1	294	.081
MFS examination	.423	1	294	.516
MFS blood	.271	1	294	.603
MFS mutilation	1.487	1	294	.224

Probability distribution: Normal
Link function: Identity
[a.] Target: donation willingness

SM Table 4. Fixed coefficients of the independent variables

					95% Confidence Interval	
	Coefficient	Std. Error	t	p	Lower	Upper
Intercept	61.005	14.689	4.153	.<0.001	32.096	89.915
Sex=1	-5.9356	4.068	-1.459	0.1456	-13.941	2.070
Sex=2	0[b]					
Age	-.460	.1288	-3.572	.000	-.714	-.207
Blood donation=1	12.785	3.6479	3.505	.001	5.606	19.965
Blood donation=2	0[b]	.	.	.	.	.
Plasma donation=1	15.021	5.1476	2.918	.004	4.890	25.152
Plasma donation=2	0[b]	.	.	.	.	.
Excluded=1	-4.778	4.2731	-1.118	.264	-13.187	3.632
Excluded=2	0[b]	.	.	.	.	.
Health status	.384	2.3715	.162	.872	-4.283	5.051
Healthcare	-.092	.2640	-.347	.729	-.611	.428
Knowledge	-.411	.6630	-.620	.536	-1.716	.894
Attitude	2.142	.2853	7.506	.000	1.580	2.703
Condition=1	-18.428	5.1791	-3.558	.000	-28.620	-8.235
Condition=2	-10.036	5.4314	-1.848	.066	-20.725	.654

Condition=3	-14.341	5.1769	-2.770	.006	-24.529	-4.152
Condition=4	.263	5.3316	.049	.961	-10.230	10.756
Condition=5	0[b]	.	.	.	.	.
MFS injection	-2.402	.7890	-3.045	.003	-3.955	-.849
MFS sharp objects	1.401	.7991	1.753	.081	-.172	2.974
MFS examination	-.376	.5776	-.650	.516	-1.513	.761
MFS blood	-.419	.8040	-.521	.603	-2.001	1.164
MFS mutilation	.654	.5365	1.219	.224	-.402	1.710

Probability distribution: Normal

Link function: Identity[a]

a. Target: donation willingness

b. This coefficient is set to zero because it is redundant.
